# Oxygen Uptake and Heart Rate Kinetics after Different Types of Resistance Exercise

**DOI:** 10.2478/hukin-2014-0077

**Published:** 2014-10-10

**Authors:** Jeferson M. Vianna, Francisco Z. Werneck, Emerson F. Coelho, Vinicius O. Damasceno, Victor M. Reis

**Affiliations:** 1 Motor Evaluation Laboratory, Faculty of Physical Education and Sports, Federal University of Juiz de Fora (UFJF) - Minas Gerais, Brazil.; 2 Federal University of Ouro Preto (UFOP) - Minas Gerais, Brazil.; 3 Federal University of Pernambuco (UFPE) – Department Physical Education, Pernambuco, Brazil.; 4 Research Centre for Sport, Health and Human Development, University of Tras-os-Montes and Alto Douro (UTAD) - Vila Real, Portugal.

**Keywords:** strength training, oxygen uptake, energy metabolism, energy expenditure

## Abstract

Oxygen uptake (VO2) and heart rate (HR) kinetics after exercise are important indicators of fitness and cardiovascular health. However, these variables have been little investigated in resistance exercise (RE). The current study compared post-exercise kinetics of VO2 and the HR among different types of REs. The study included 14 males (age: 26.5±5.4 years, body mass: 80.1±11.4 kg, body height: 1.77±0.07 m, fat content: 11.3±4.6%) with RE experience. Dynamic muscle strength was measured using one repetition maximum (1RM) with regard to the half-squat, bench press, pull-down, and triceps pushdown exercises. The participants performed a maximum number of repetitions at 80% of 1RM for each exercise, separated by a recovery period of 60 minutes. VO2 was measured using ergospirometry. VO2 and HR kinetics were assessed using the time constant of the recovery curves, and excess oxygen consumption (EPOC) was calculated afterward. Significant differences were not observed across the exercises with regard to VO2 kinetics. However, the half-squat exercise elicited a greater EPOC than the bench press and triceps pushdown exercises (p<.05). HR kinetics was slower for the half-squat exercise than for the other exercises (p<.05). These findings confirm that the type of RE influences both the cardiac autonomic response post-exercise and EPOC, but not VO2 kinetics.

## Introduction

Resistance exercise (RE) causes positive cardiovascular and metabolic alterations. Furthermore, RE is currently one of the most common forms of weight control, and improves fitness and health (ACSM, 2009). Physiological adaptations can be quantified by studying the heart rate (HR) and oxygen consumption (VO2) kinetics during and after exercise (Almeida et al., 2011; Buitrago et al., 2011; Kacin and Strazar, 2011; Heffernan et al., 2006; Short and Sedlock, 1997).

RE has been extensively studied with regard to energy costs by measuring VO2 (Vianna et al., 2011; Pinto et al., 2011; Robergs et al., 2007; Bloomer, 2005; Phillips and Ziuraitis, 2003; Schuenke et al., 2002). After an acute RE session, VO2 does not return to resting values immediately; the energy expenditure remains higher than the basal metabolism for a while. This “extra” energy demand during the recovery period is called “excess oxygen consumption after exercise” (EPOC) (Gaesser and Brooks, 1984). Reviews on this subject indicate that the magnitude and duration of EPOC are directly proportional to the intensity of exercise (Castinheiras Neto et al., 2009; Meirelles and Gomes, 2004); however, the effect of different types of RE on EPOC has not been investigated.

RE causes significant cardiovascular stress (Collins et al., 1991) and changes in cardiac autonomic balance (Lima et al., 2011; Heffernan et al., 2006). Both VO2 and the HR decrease quickly after exercise due to a decreased demand for oxygen from the muscles and the reactivation of vagal tone (Imai et al., 1994). VO2 and HR kinetics are evaluated as the time required for these variables to return to equilibrium after exercise by measuring the exponential time constant of the tau (τ) curves (Jones and Poole, 2005). Higher tau values imply slower kinetics and are associated with functional impairments and a higher risk of mortality (Jones and Poole, 2005; Cole et al., 1999).

Therefore, VO2 and HR kinetics after exercise are important indicators of physical fitness and cardiovascular health (Sietsema et al., 1989). However, the behaviour of these variables with regard to RE has received little research attention. VO2 kinetics has been studied in aerobic exercises (Jones and Burnley, 2005). With regard to RE, a recent study by Almeida et al. (2011) did not find significant differences in VO2 kinetics between sessions studying hypertrophy versus local muscular endurance. Recovery oxygen uptake in response to two resistance training sessions at different intensities demonstrated that a RE session with the aim of LME gain is capable of causing similar metabolic impact to the RE session with the hypertrophy aim, even if it was performed at lower intensity concerning maximal load.

Understanding the adjustments of metabolism and the cardiovascular system to the different types of RE during recovery might allow researchers to safely prescribe exercises and structure training programs that maximise energy expenditure. Thus, the current study compared post-exercise VO2 and HR kinetics across different REs. Given the available literature, we hypothesised that exercises involving larger muscle mass would have greater EPOC and slower kinetics during recovery.

## Material and Methods

### Participants

The participants were recruited intentionally among a population of adults with previous non-competitive experience in resistance exercise. The selected volunteers included 14 men (age: 26.5±5.4 years, body mass: 80.1±11.4 kg, body height: 1.77±0.07 m, fat content: 11.3±4.6%) who were in good health and had trained as bodybuilders for at least one year, with a weekly training volume equal to or exceeding three sessions. The exclusion criteria for the study were the use of medications that influenced the stress response, the presence of acute diseases, or any history of neuromuscular injury. The Ethics Committee for Research of the Universities Trásos-Montes and Alto Douro approved the experimental protocol, and written consent was obtained from all subjects. The study complied with Resolution 196/96 of the National Health Board on human research.

### Experimental Protocol

**Figure 1:**
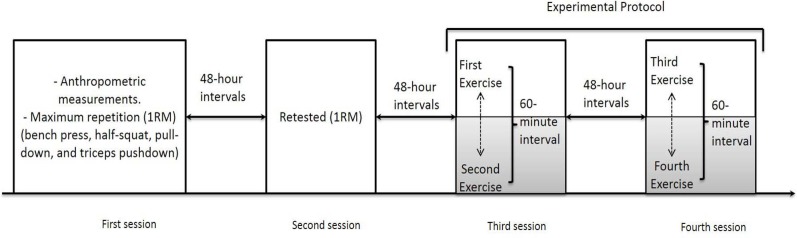
Experimental protocol - design

Each participant completed four sessions in the gym for data collection. On the first visit, anthropometric measurements were taken, and the one repetition maximum (1RM) test was performed for four selected exercises: bench press, half-squat, pull-down, and triceps pushdown. Participants were retested at the second visit. On the third and fourth visits, the participants performed 80% of 1RM intensity tests in which the type and order of the exercises were chosen at random. All sessions were conducted in the afternoon at 48 hour intervals, at a room temperature of 20–25 degrees and 35–45% relative humidity (Figure 1).

### Anthropometric Assessment

Body mass was measured using an electronic scale (Tanita Corporation, BC-531, Tokyo, Japan) with accuracy of 0.1kg. Body height was measured using a tape measure (“Misura per salti”) with accuracy of 1 mm. An experienced assessor measured fat mass using skinfolds with a Lange® lipo-calliper (Cambridge Scientific Industries, Cambridge, MD). The following anatomical sites were measured: pectoral, midaxillary, triceps, subscapular, abdominal, suprailiac, and thigh. The Jackson and Pollock (1978) formula was used to obtain body density, and the Siri Equation was applied to convert density into fat mass.

### Dynamic Muscle Strength Evaluation

Muscle strength was measured using the dynamic 1RM test across the following exercises: bench press, half free weight squats, pull-down, and triceps pushdown. The following procedures were used to perform the 1RM tests: 1) general activation with five to ten repetitions and a load between 40% and 60% of the maximum, followed by 1 minute of stretching; 2) three to five repetitions with a load between 60% and 80% of the maximum load, followed by a 2 min rest period; and 3) one attempt at the maximum load. After the participants succeeded or failed at the maximum load, they were allowed to rest for 5 minutes, and the load was increased or decreased for the next attempt. The maximum load (kg) was designated as the load at which participants were able to perform a single repetition out of five attempts. A retest was performed 48 hours later to assess test reliability, and the largest load from both days (test and retest) was considered when participants showed less than a 5% difference. Participants with greater differences were required to appear at the test site again to perform additional tests and subsequently the differences between test sessions were calculated. Between-test exercises that might interfere with the results during the 48 hours period were not allowed. A professional with experience in resistance training always accompanied the participants. The final 1RM loads for the exercises were as follows: bench press = 95.3±22.4 kg; half squat = 124.1±33.9 kg; pull-down = 94.7±13.4 kg; and triceps pushdown = 48.2±10 kg.

### Exercise session

Before each exercise session, a general warm-up for 10 minutes was accomplished on a cycle ergometer with a load equivalent to 2% of participant body mass. Then, the participants performed exercises for joint mobility. After warming up, the participants remained seated and were equipped with an HR monitor and a mask to collect expired air, coupled to a gas analyser. Participants remained at rest for 5 minutes. After the rest period, the RE session was restarted at an intensity of 80% of 1RM, for the bench press, half-squat, triceps pushdown, and pull-down exercises. The execution order of the various exercises was random, and the exercises were performed across two sessions at 48 hour intervals. All exercises were performed on Panatta Sport (Italy) brand equipment. Immediately after the exercises, the participants remained seated in a chair during the first 5 min of the recovery period. An electronic metronome was used to control the rate of exercises performed, with 2 s for the eccentric phase and 1 s for the concentric phase (40 bpm; 20 repetitions per minute).

### VO_2_ and HR Measurement

During the exercise session, VO_2_, carbon dioxide production, and ventilation were measured using a portable open-circuit gas-exchange analyzer (COSMED K4b^2^, *Rome, Italy*). The equipment was calibrated for ambient air, reference gases, time delay, and turbine before each study session. After completing each exercise set, the participants remained seated in a chair for 5 min, and the data for the recovery period were collected. HR was measured continuously during all exercise sessions via a portable monitor *(Polar Wireless Double Electrode; Kempele, Finland).* The participants remained in the environment until the next exercise.

### VO2 and HR kinetics measures during recovery

VO_2_ kinetics was assessed after performing the REs from a mean of 10 s in the breath-to-breath data. All values recorded during 5 min of the passive recovery between each exercise were used. Peak VO_2_ (VO_2peak_) was defined as the highest VO_2_ value at the end of each session, and this value was the starting point of the recovery protocol. After finishing the exercise, VO_2_ decreased exponentially. Thus, the VO_2_ time recovery constant (τVO_2_) was determined by fitting a monoexponential curve. The τVO_2_ represents the time required to reach 63% of peak VO_2_, and this value was calculated using a Levenberg-Marquardt least squares nonlinear regression via MATLAB. The general equation was VO_2_(t)=Ae^-kt^+C (Cohen-Solal et al., 1995), where VO_2_(t) was VO_2_ at time t, A was the VO_2_ response amplitude during recovery, K was the constant decrease in VO_2_ (K^-1^=τVO_2_), and C was the VO_2_ value in the 5th minute of recovery. The same model was used to calculate HR kinetics.

### EPOC Fast Component Calculation

The EPOC fast component was determined using the following formula: EPOC_(L/min)_=A*τVO_2_, where A was the VO_2_ amplitude of recovery variation and τ was the time constant.

### Data Analyses

The data from the descriptive analysis are presented as means ± standard deviations. A repeated-measures ANOVA was used to test for differences in the EPOC fast component as well as the VO_2_ and HR kinetics with regard to the REs when the assumptions of normality and homogeneity of variance were met. The Huynh-Feldt Epsilon was used to correct for the violated sphericity assumption. Multiple mean comparisons were performed using the Bonferroni correction when significant differences were detected. The time constants were compared using paired-samples t-tests. Pearson’s correlation was used to examine the associations among variables. All data were analysed using SPSS version 16.0 at the level of significance set at p<0.05.

## Results

This study did not find significant differences in pre-exercise VO_2_ (p>.05), indicating that individuals started with similar VO_2_ values. Likewise, significant differences were not observed with regard to the time taken to perform the exercises (F_3,39_=0.490, p=.69). However, significant differences were observed for the number of repetitions (F_3,39_=3.395, p=.03) and total weight lifted (F_3,39_=17.847, p=.001). A multiple mean comparison showed that participants repeated fewer bench press exercises compared with pull-down or triceps pushdown exercises (p<.01); however, significant differences were not observed for the half-squat exercise. Higher total weight lifted was observed for the half-squat and pull-down exercises (p<0.01; Table 1).

### VO2 and EPOC kinetics

Significantly different VO_2_ values occurred between exercises at the exercise peaks (F_3,39_=32.387, p=0.001; Table 2).

**Figure 2:**
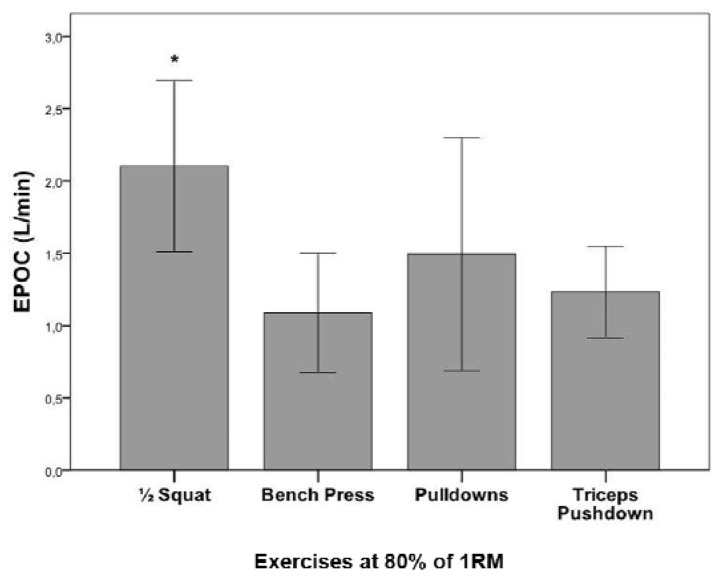
Mean ± standard deviation of the EPOC (excess of post-oxygen consumption) after one set of different types of resistance exercise at 80% of 1RM. (* Significant differences between the bench press and triceps pull down, p<0.05).

**Figure 3:**
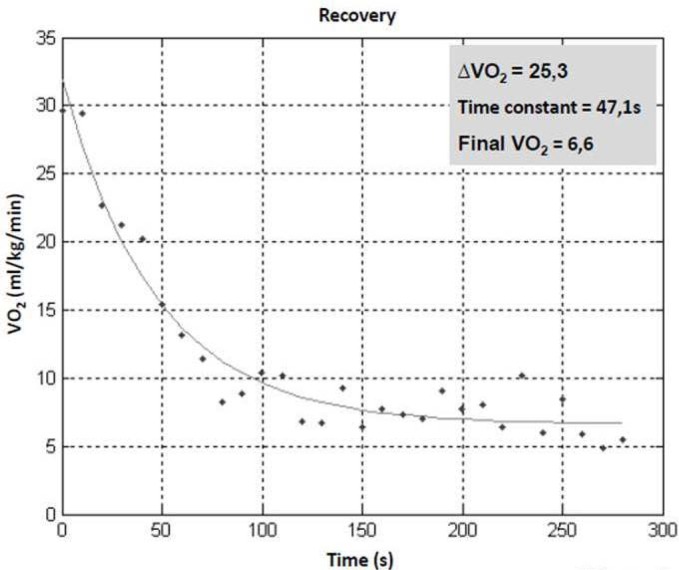
Representative example of VO_2_ kinetics after resistance exercises (half squat, bench press, pull down and triceps pull down) with intensity of 80% of 1RM.

The highest VO_2peak_ values were observed after participants performed the half-squat, followed by the pull-down and triceps pushdown exercises; these values were all higher than the VO_2_ observed for the bench press (p<.05). The same result was observed for ΔVO_2_ (Table 2). At the end of the 5th minute of recovery, participants’ VO_2_ values returned to pre-exercise levels regardless of the type of exercise performed (F_3,39_=.040, p=.99). Significant differences were not observed with regard to the VO_2_ kinetics among the exercises analysed (F_3,39_=.924, p=.44; Figure 2) after considering the entire recovery period. A significant difference was observed among the exercises with regard to the EPOC (F_3,39_=9.587, p<.001). The EPOC was higher for the half-squat than for the bench press and triceps pushdown exercises, although no significant differences were observed for the pull-down exercise (p<.05). No significant differences were observed with regard to the EPOC for the bench press, pull-down, or triceps pushdown exercises (Figure 3).

### HR Kinetics

No significant differences were observed among the exercises with regard to the HR at exercise peak (F_3,39_=1.033, p=.38; Table 2). However, the variation in the HR amplitude during recovery was greater for the half-squat and bench press exercises compared with the pull-down and triceps pull-down exercises (F_3,39_=3.562, p=.04). At the end of the 5th minute of recovery, the HR was lower for the half-squat and bench press exercises than for the pull-down or triceps pushdown exercises (F_3,39_=15.514, p=.001; Table 2).

**Figure 4:**
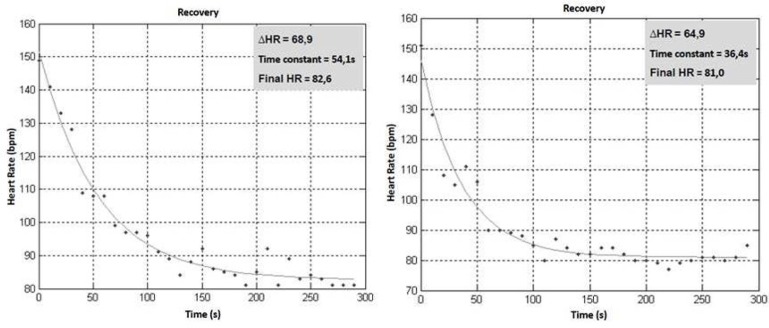
Representative example of heart rate kinetics after resistance exercises of half squat (left panel), bench press, pull down and triceps pull down (right panel) with intensity of 80% of 1RM.

Significant differences were found during recovery with regard to the τHR (F_3,39_=5.174, p=.01; Figure 4). The half squat exercise showed a greater τHR compared with the other exercises (p<.05). The decrease in the HR after the first minute of recovery was similar across the exercises analysed. On average, individuals reduced the HR by 55 bpm during the first minute of recovery. Significant differences were not found between the τHR and τVO_2_ regarding the exercises analysed. Furthermore, significant correlations were not found among VO_2_, HR kinetics, or the variables related to exercise intensity (i.e., tonnage, exercise duration, number of repetitions, and tonnage/time); the same result was true for the individuals’ experience and the 1RM strength.

## Discussion

This study showed that the type of exercise performed until exhaustion influenced the metabolic and autonomic responses during recovery from RE to 80% of 1RM. From a metabolic point of view, the type of exercise did not influence VO_2_ kinetics during recovery, but the EPOC was higher for the half-squat compared with the bench press and triceps pushdown exercises (which are similar to the pull-down exercise). Regarding autonomic modulation, slower HR kinetics was observed during the half-squat than during the other exercises, which implies an attenuated vagal reactivation. This finding indicates that the EPOC and vagal reactivation are directly and inversely proportional, respectively, to the mass involved in RE.

This study is the first to investigate the effect of the type of exercise (i.e., influence of muscle groups) on VO_2_ and the HR kinetic response. In aerobic exercise, VO_2_ kinetics of the upper limbs is slower than those of the lower limbs (Jones and Burnley, 2005); however, the amount of muscle mass involved during the activity does not influence VO_2_ response kinetics (Koga et al., 2001). This study showed that the type of RE performed did not influence the VO_2_ decline rate (i.e., free weight vs. machine; upper limbs vs. lower limbs; small muscle mass vs. large muscle mass). Excluding the half squat (which showed a slightly higher time constant), the VO_2_ decrease after RE was 47 s, regardless of VO_2peak_. High VO_2_ kinetic values (e.g., typically over 70) characterise patients with cardiovascular system impairments (Cohen-Solal et al., 1995). Our results match those of Almeida et al. (2011), who were also unable to find a difference in VO_2_ kinetics between sessions of local muscular strength and hypertrophy. On the other hand, Thornton and Potteiger (2002) suggested that VO_2_ behaviour in the fast phase was the difference between the EPOC values in strength training sessions.

The influences of the greater demand and performance of the stabilising and synergist muscles during the pull-down and triceps pushdown exercises might explain our results. The physiological mechanisms involved in the delayed VO_2_ kinetic response remain controversial in the literature. VO_2_ declines rapidly soon after completing exercise; the rate of this decline is likely related to phosphocreatine re-synthesis and the stock of myoglobin O_2_ (Tschakovsky and Hughson, 1999). Certain factors influence this process, such as exercise intensity (Koppo et al., 2004), physical fitness (Short and Sedlock, 1997; Koppo et al., 2004), and lactate production (Xu and Rhodes, 1999).

After exercise but during the period characterised by the EPOC, the blood lactate that accumulated during exercise is oxidised, the O_2_ reserves in haemoglobin and myoglobin are restored, and the reserves of adenosine triphosphate (ATP) and phosphocreatine (PC) in the muscles and glycogen deposits return to normal. Cardiorespiratory function remains high so that sufficient oxygen is provided to tissues, thereby enabling the organism to re-establish its baseline. A fast component exists between 25 s and 30 s associated with phosphocreatine re-synthesis and the restoration of the muscular O_2_ stock, followed by a slow component for several minutes to compensate for the O_2_ deficits due to the accumulation of lactate at the beginning of exercise (Gaesser and Brooks, 1984). This aerobic exercise knowledge is current; however, little is known regarding the mechanisms responsible for VO_2_ kinetics during RE.

The metabolic and hemodynamic response in the present study corroborates the findings of Collins et al. (1991). These authors reported average heart rate of 161bpm and average VO_2_ of 1.86 L/min after RE performed at 70% of 1RM and found a greater EPOC at this intensity, when compared to lower intensities. MacDougall et al. (1988) found that a single set of 10RM caused decreases of 57% in phosphocreatine, of 13% in glycogen and of 29% in ATP, as well as post-exercise lactate of 16 mmol/kg. Interestingly, the EPOC caused by pull-down and half-squat exercise was similar. This similarity may be explained by the involvement of the muscle groups used in body stabilisation during the pull-down exercise, despite the apparent larger muscle mass involved in the half-squat.

Regarding HR kinetics, vagal reactivation was slower after exercising the lower limbs compared with the upper limbs. Assessing HR kinetics is one way to measure vagal reactivation after exercise. Few studies have evaluated HR kinetics after RE; however, the inverse relationship between the metabolic stress of anaerobic activity and HR recovery (Buchheit et al., 2007) is consistent with the results of the present study. Due to the cardiovascular risk associated with the magnitude of post-exercise vagal reactivation (Cole et al., 1999) and the relationship between the size of the muscle groups and the cardiovascular stress of an exercise, individuals with high cardiovascular risks should most likely not perform the half-squat exercise.

Studying HR recovery is of paramount importance with regard to diagnostic and prognostic possibilities of different diseases (Cole et al., 1999). Given the clinical importance and possible use of this information with regard to exercise prescription, the RE variables that influence HR kinetics must be investigated. The autonomic imbalance caused by RE is greater than that caused by aerobic exercise; the former promotes a slower HR recovery after the first period than the latter (Heffernan et al., 2006). Additional studies should be performed on this population to support or refute our findings. On the other hand, if an individual’s goal is weight control, then exercises that involve the major muscle groups should be priority.

This study was limited by its small sample size and the type of the protocol used, as it does not necessarily represent a normal training format. The participants were apparently athletic and healthy youth men with normal body size and body fat content. Women and elderly people may response differently and obese individuals should be considered as a separate group for this kind of studies to investigate their responses. Additional studies should be performed to compare the common and daily training protocols of participants and measure their lactate levels after RE.

## Conclusions

The current findings confirm that RE influences the cardiac autonomic response post-exercise as measured via HR kinetics and the EPOC but not via VO_2_ kinetics. VO_2_ dynamic behaviour during recovery after RE was similar across the different types of exercise regardless of the muscle mass involved, whereas HR kinetics was slower for exercises that involved more muscle mass.

## Figures and Tables

**Table 1:** Mean ± standard deviation of the time of exercise, number of repetitions and total weight lifted of resistance exercise with intensity of 80% of 1RM (n=14)

Variable	Half squat	Bench Press	Pull down	Triceps pull down
Time (s)	28.2 ± 11.4 ^a^	26.4 ± 11.4 ^a^	29.4 ± 3.6 ^a^	28.8 ± 8.4 ^a^
Repetitions (reps)	11.4 ± 3.1 ^a, b^	8.7 ± 1.9 ^a^	11.1 ± 1.6 ^b^	11.4 ± 3.7 ^b^
Total weight lifted (kg)	1088 ± 48 ^a^	642 ± 206 ^b^	821 ± 177 ^a^	421 ± 171 ^c^

(Different letters in the same row indicate significant differences; p<0.05).

**Table 2:** Mean ± standard deviation of the responses post-exercise VO_2_ and HR kinetics in resistance exercise of the intensity at the level of 80% of 1RM (n = 14).

Variable	Half squat	Bench press	Pull down	Triceps pull down
VO_2pico_ (ml/kg/min)	31,3 ± 6,4^a^	17,3 ± 3,5^b^	22,9 ± 5,2^c^	22,1 ± 2,8^c^
ΔVO_2_ (ml/kg/min)	24,8 ± 6,0^a^	10,7 ± 3,5^b^	16,3 ± 5,2^c^	15,6 ± 2,8^c^
VO_2rec_ (ml/kg/min)	6,4 ± 1,1	6,5 ± 1,1	6,5 ± 0,90	6,5 ± 1,2
τVO_2_ (s)	51,9 ± 14,0	46,9 ± 12,9	46,8 ± 13,9	43,0 ± 12,8
EPOC (L/min)	2,10 ± 0,59^a^	1,09 ± 0,41^b^	1,50 ± 0,80ª^,b^	1,23 ±0,32^b^
HR_pico_ (bpm)	159 ± 15	162 ± 30	151 ± 19	159 ± 16
HR_rec_ (bpm)	80,7 ± 10,8^a^	81,8 ± 10,6^a^	88,2 ± 12,6^b^	89,8 ± 11,0^b^
τHR (s)	58,8 ± 16,8^a^	38,0 ± 18,8^b^	39,2 ± 18,8^b^	36,6 ± 16,5^b^

(Different letters in the same row indicate significant differences; p<0,05). VO_2rec_ = recovery in 5^º^ min. HR_rec_= recovery in 5^º^ min.
